# Deep learning-based dental age estimation in adolescents using panoramic radiographs: a comparative study with traditional methods

**DOI:** 10.3389/fradi.2026.1754772

**Published:** 2026-05-13

**Authors:** Taseer Bashir, Hanadi Abdullah Alwafi

**Affiliations:** 1 Department of Oral Medicine and Radiology, Batterjee Medical College, Jeddah, Saudi Arabia; 2 Basic and Preventive Sciences Department, Batterjee Medical College (BMC), Jeddah, Saudi Arabia

**Keywords:** artificial intelligence, deep learning, dental age estimation, forensic odontology, panoramic radiology

## Abstract

**Introduction:**

Accurate dental age estimation is a vital component of forensic identification, orthodontic treatment planning, and medico-legal assessments. Conventional approaches, such as Demirjian's and Cameriere's methods, are limited by observer variability and population dependency. Recent advances in artificial intelligence (AI) and deep learning (DL) offer potential for objective, reproducible, and scalable assessment through panoramic radiology. This study aimed to develop and validate a convolutional neural network (CNN)-based model for dental age estimation among adolescents using panoramic radiographs and to compare its accuracy with traditional manual methods.

**Methods:**

A total of 296 panoramic dental radiographs from adolescents aged 9–19 years were analyzed, with balanced representation across age groups and comparable distribution between male and female participants. Images were divided into training (70%, *n* = 207), validation (15%, *n* = 44), and testing (15%, *n* = 45) subsets. All images were preprocessed, normalized, and augmented prior to model development. A CNN-based regression model was trained using mean squared error as the loss function and evaluated using mean absolute error (MAE), root mean square error (RMSE), and Pearson correlation coefficient (r). Grad-CAM visualization was employed to enhance interpretability of model predictions.

**Results:**

The proposed CNN model achieved an MAE of 1.12 years, RMSE of 1.37 years, and a strong correlation (r = 0.96) between predicted and chronological age, outperforming traditional Demirjian and Cameriere methods. Activation maps confirmed that predictions were based on biologically relevant anatomical regions, including developing third molars and mandibular growth zones.

**Conclusion:**

AI-driven panoramic radiographic analysis provides a robust, interpretable, and ethically compliant framework for adolescent dental age estimation. These findings highlight its translational potential in forensic odontology, pediatric dentistry, and legal age verification, supporting a shift toward standardized, data-driven dental diagnostics.

## 1 Introduction

Accurate estimation of chronological age using dental radiographic evidence remains a cornerstone of forensic identification, orthodontic treatment planning, and medico-legal decision-making. Dental tissues, unlike many other biological indicators, are highly resistant to environmental and post-mortem changes and provide reliable developmental markers across childhood and adolescence. The predictable sequence of tooth formation, calcification, and eruption allows clinicians and forensic experts to approximate age even in the absence of reliable documentation. Among available imaging modalities, panoramic radiography (orthopantomography) offers a comprehensive two-dimensional visualization of the maxilla and mandible, capturing dentition and surrounding structures in a single image (1). Its accessibility, low radiation exposure, and diagnostic breadth have made it the preferred modality for dental age estimation, particularly during adolescence—a critical period characterized by rapid and heterogeneous dental development.

Traditional dental age estimation techniques, such as Demirjian's staging system and Cameriere's open-apex method, have long served as standard approaches in forensic odontology. Demirjian's method evaluates the developmental stages of mandibular teeth and assigns maturity scores based on predefined criteria, while Cameriere's method quantifies the degree of root development by measuring the width of open apices relative to tooth length. Although these techniques have been widely validated and applied across populations, they are inherently limited by reliance on manual interpretation, inter- and intra-observer variability, and the need for population-specific calibration. Their performance may also decline during late adolescence due to overlapping developmental stages and increased biological variability. Atlas-based methods, such as the London Atlas of Tooth Development and Eruption, (2), further standardize visual assessment but remain dependent on subjective interpretation and regional applicability (3). As global mobility and diverse population structures continue to expand, there is cumulative mandate for data-driven, impartial, and reproducible techniques that minimize human bias and accommodate ethnic variability.

In recent years, artificial intelligence (AI) and machine learning (ML) techniques have significantly advanced the field of medical and dental image analysis. Deep learning (DL), particularly convolutional neural networks (CNNs), has demonstrated remarkable capability in extracting complex hierarchical features from radiographic images without the need for handcrafted descriptors. These models have been successfully applied in dentistry for tasks such as caries detection, lesion identification, tooth segmentation, and orthodontic assessment (4). Extending these approaches to dental age estimation enables automated quantification of morphological and developmental changes that may not be readily discernible to human observers. Early studies have demonstrated the feasibility of CNN-based models in predicting age from panoramic radiographs, showing improved performance compared to traditional morphometric approaches and strong correlations between predicted and chronological age.

Subsequent research has focused on enhancing predictive performance through advanced deep learning architectures and transfer learning strategies. Models based on ResNet, Xception, EfficientNet, and hybrid CNN frameworks have achieved mean absolute errors (MAE) approaching or even below one year in some studies, highlighting the increasing maturity of AI-based dental age estimation. Transfer learning, in particular, has enabled effective utilization of pre-trained models to overcome limitations associated with smaller medical datasets. Firstly, CNNs were used by Wallraff et al. (2021) (5) to predict age in dental x-ray images, and it was demonstrated that the learned image features were capable of more effectively capturing developmental gradients compared to handcrafted morphometric measures. Based on these premises, Lee et al. (6) used machine-learning algorithms on panoramic radio morphometric parameters and obtained significant correlations between predicted and chronological ages.

Studies such as those by Bizjak and Robič and Şahin and Kölüş have demonstrated that AI models can achieve high accuracy while simultaneously extending applications to multi-task learning scenarios, including combined age and sex estimation (7), DentAge was proposed as a DL model, featuring a mixture of convolutional and fully connected layers of regression, with mean absolute errors (MAEs) of nearly one year, which was assessed on independent data sets. On the same note, Şahin and Kölüş (8) extended the use of panoramic-based CNNs to estimate age and sex simultaneously, thus increasing the forensic usability of AI in population recognition.

Beyond conventional deep learning approaches, recent research has explored hybrid and surrogate modeling techniques that integrate domain-specific anatomical knowledge with advanced statistical learning frameworks. Notably, Zaborowicz et al. (9) have introduced innovative approaches that combine deep learning with reduced-order modeling methods, such as Proper Orthogonal Decomposition (POD) coupled with Gaussian Process regression (POD-GP) (10). These hybrid frameworks aim to balance predictive accuracy with computational efficiency and interpretability by reducing the dimensionality of complex imaging data while preserving essential morphological features. Such approaches represent an important evolution in dental age estimation, bridging the gap between purely data-driven models and physiologically meaningful representations of dental development. By incorporating both geometric descriptors and machine learning techniques, these models enhance generalizability and provide insights into the underlying biological processes governing tooth maturation.

Despite these important advancements, current research still lacks a unified framework that simultaneously addresses predictive accuracy, reproducibility, and interpretability. While deep learning models have achieved high performance, they often rely on proprietary datasets and function as black-box systems. Similarly, hybrid approaches, although promising, remain computationally complex and are not widely validated across standardized open datasets. This creates a gap between methodological innovation and practical clinical or forensic applicability. Moreover, existing studies often evaluate models in isolation without standardized benchmarking against traditional forensic methods, further limiting the ability to assess clinical applicability and real-world relevance.

Compared to hybrid modeling approaches such as POD-GP, which focus on dimensionality reduction and computational efficiency, the present study emphasizes clinical interpretability and reproducibility using standardized imaging data, thereby complementing rather than competing with such methodologies.

In addition to improvements in predictive accuracy, interpretability has emerged as a critical requirement for clinical and forensic applications of AI. While deep learning models can achieve high performance, their “black-box” nature often limits acceptance in medico-legal contexts where transparency and explainability are essential. Techniques such as Gradient-weighted Class Activation Mapping (Grad-CAM) provide a mechanism for visualizing model attention by highlighting regions of the image that contribute most significantly to the prediction. In the context of dental age estimation, Grad-CAM has been used to demonstrate that AI models focus on biologically relevant anatomical regions, including developing third molars, crown calcification zones, and mandibular growth areas. Such interpretability not only enhances confidence in model predictions but also ensures alignment with established principles of odontogenesis (11).

Despite these advancements, several challenges remain in the current research landscape. Many studies rely on proprietary or institution-specific datasets, limiting reproducibility and hindering independent validation. The lack of standardized, publicly available datasets makes it difficult to compare models across studies and assess their true generalizability. Furthermore, the use of mixed-age cohorts often obscures performance variability during adolescence—a critical medico-legal period where accurate age estimation is particularly important. Variations in imaging protocols, resolution, and contrast across institutions also introduce challenges in model transferability. Additionally, while more complex architectures and hybrid models have improved accuracy, they often increase computational complexity and reduce interpretability, creating a trade-off between performance and transparency.

These limitations underscore the need for reproducible, transparent, and clinically interpretable AI frameworks for dental age estimation. In this context, the present study is positioned as a comprehensive and reproducible approach within the evolving field of AI-driven forensic odontology. The study focuses specifically on adolescents aged 9–19 years, a group characterized by significant dental developmental changes and high medico-legal relevance. A fully open-access panoramic radiograph dataset is utilized to ensure transparency and enable independent replication. Both a custom CNN model and a transfer learning approach using ResNet50 are developed and evaluated under identical conditions. Importantly, the study includes direct benchmarking against traditional Demirjian and Cameriere methods, providing a clear comparison between manual and AI-based approaches.

Furthermore, the integration of Grad-CAM visualization enhances interpretability by allowing assessment of whether model predictions are based on anatomically meaningful regions. In this context, the present study is positioned not as a purely architectural innovation but as a reproducible and interpretable validation framework within the evolving research landscape. Unlike many prior studies, this work integrates three key components: (1) the use of a fully open-access dataset to ensure transparency and reproducibility, (2) direct benchmarking against established forensic methods (Demirjian and Cameriere), and (3) incorporation of explainable AI through Grad-CAM visualization. This integrated approach addresses critical gaps in current literature by combining performance evaluation with clinical interpretability and methodological transparency.

The present study aims to provide a robust and clinically relevant framework for dental age estimation by integrating panoramic radiography with advanced deep learning techniques. Specifically, the objectives are threefold: (1) to develop and validate an AI-based model for estimating dental age in adolescents, (2) to compare its performance with traditional estimation methods, and (3) to evaluate the interpretability of AI predictions using visual activation mapping techniques. It is hypothesized that AI-based panoramic radiology can achieve greater accuracy, consistency, and objectivity compared to conventional human-based assessment methods.

## 2 Materials and methods

### 2.1 Study design

The research was a cross-sectional, observational study that sought to develop and validate an AI-based model to predict the sequential age of adolescents using panoramic dental radiographs. The study was done according to the ethical principles of secondary data analysis of anonymized medical images. The research process involved five consecutive stages (1) data collection and preparation, (2) labelling, (3) dataset preparation and division, (4) AI model construction and training, and (5) result comparison to conventional estimation criteria.

### 2.2 Data source and characteristics

The dataset used in this study was obtained from a publicly available open-access repository hosted on Zenodo (Archive ID: 16745408.zip). The dataset consists of panoramic dental radiographs curated for age estimation research purposes. It includes 296 images in JPG format representing individuals aged 9–19 years. The dataset is associated with a unique Digital Object Identifier (DOI) and complies with open scientific data standards, enabling reproducibility and transparency. Detailed metadata, including image characteristics and age annotations, are provided within the repository.

### 2.3 Data labeling

Age annotations were extracted from the dataset's labeling structure and embedded metadata using a standardized parsing protocol, and subsequently verified for consistency. A structured dataset was then created linking each panoramic radiographic image to its corresponding chronological age and dataset split (training, validation, and testing). This approach ensured accurate labeling, complete supervision for model training, and maintained methodological transparency.

### 2.4 Data preprocessing

All images were resized to 256 × 256 pixels, converted to grayscale where applicable, and intensity-normalized by scaling pixel values to the range (0,1). Data augmentation included random rotation (±15 degrees), horizontal flipping, brightness variation (±20%), and minor zoom adjustments to enhance generalization.

### 2.5 Model architecture

A convolutional neural network (CNN) architecture was developed for regression-based age estimation.

The baseline model structure consisted of:

- **Input Layer:** 256 × 256 × 3 RGB images.
- **Convolutional Layers:** Three convolutional blocks (3 × 3 filters) with ReLU activations and max-pooling layers.
- **Fully Connected Layers:** Two dense layers (128 and 64 neurons) followed by dropout (0.3) to prevent overfitting.
- **Output Layer:** A single neuron with linear activation predicting continuous age values.

#### 2.5.1 Transfer learning (ResNet50 model)

In addition to the custom CNN architecture, a transfer learning approach using ResNet50 was implemented to enhance predictive performance. The ResNet50 model was initialized with pre-trained ImageNet weights, and the final classification layers were replaced with fully connected regression layers to predict continuous age values. Initially, the convolutional base was frozen to retain learned feature representations, followed by partial unfreezing of deeper layers for fine-tuning. The model was trained using the same dataset splits, preprocessing pipeline, optimizer (Adam), learning rate (0.001), and batch size (16) as the CNN model to ensure fair comparison. Performance evaluation metrics included MAE, RMSE, and correlation coefficient (r).

### 2.6 Model training

- **Loss Function:** Mean Squared Error (MSE).
- **Optimizer:** Adam optimizer with a learning rate of 0.001.
- **Batch Size:** 16 images per batch.
- **Epochs:** 50, with early stopping based on validation loss.
- **Evaluation Metrics:** MAE, Root Mean Square Error (RMSE), and coefficient of determination (R²).

### 2.7 Model evaluation

The model's predictive accuracy was assessed using the test set (15%) and compared with traditional age estimation techniques (Demirjian and Cameriere). Statistical analyses included:

- **MAE and RMSE** for prediction error quantification.
- **Pearson correlation (r)** between predicted and actual ages.
- **Bland–Altman analysis** for agreement assessment between AI and traditional methods.
- **t-tests** to evaluate differences in mean prediction errors.

Performance benchmarks for acceptability were defined as MAE < 1.5 years and R² > 0.90, based on prior literature in dental AI research.

#### 2.7.1 Implementation of traditional age estimation methods

For comparative analysis, two widely used forensic dental age estimation techniques—Demirjian's and Cameriere's methods—were applied to the same dataset. Demirjian's method involves assigning developmental stages (A–H) to selected mandibular teeth, which are then converted into maturity scores and corresponding age estimates based on established reference tables. Cameriere's method estimates age by measuring the degree of root development, specifically the width of open apices relative to tooth length, followed by regression-based age calculation. Both methods were implemented following standard published protocols to ensure methodological consistency and enable fair comparison with AI-based models.

### 2.8 Ethical considerations

This study qualified for Institutional Review Board (IRB) exemption as it involved secondary analysis of publicly available, fully anonymized data from the Zenodo repository (Archive ID: 16745408.zip). No personal identifiers were accessible, and informed consent was waived in accordance with ethical guidelines.

This study utilized anonymized, publicly available panoramic radiographs containing no personal identifiers. All data were used solely for academic purposes in accordance with ethical standards for research involving secondary datasets, ensuring confidentiality and responsible data handling.

## 3 Results

### 3.1 Dataset overview

A total of 296 panoramic radiographs were evaluated, covering adolescent subjects aged 9–19 years. All images were of diagnostic quality and suitable for feature extraction.

The dataset was partitioned into working out (*n* = 207; 70%), justification (*n* = 44; 15%), and testing (*n* = 45; 15%) subsets, maintaining balanced age and sex distributions (Table 1).

**Table 1:** Dataset distribution by age group.

Age (Years)	Number of Images	Percentage (%)
9–10	38	12.8
11–12	55	18.6
13–14	61	20.6
15–16	67	22.6
17–19	75	25.4
**Total**	**296**	**100** **.** **0**

A Total of 196 panoramic radiographs being evaluated covering subjects from 9–19 years showing balanced distribution.

**Figure 1:**
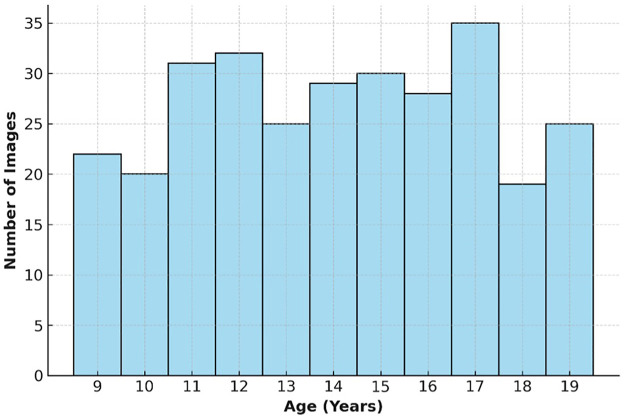
Age distribution histogram of the dataset (ages 9–19).

Figure 1 shows the age-wise histogram of the dataset, demonstrating uniform representation across adolescence.

### 3.2 Image preprocessing and quality enhancement

All panoramic radiographs were normalized to 256 × 256 pixels, grayscale converted, and contrast-enhanced using histogram equalization. Data augmentation yielded an effective training set of approximately 1,242 images after random rotation, flipping, and brightness scaling.

**Figure 2:**
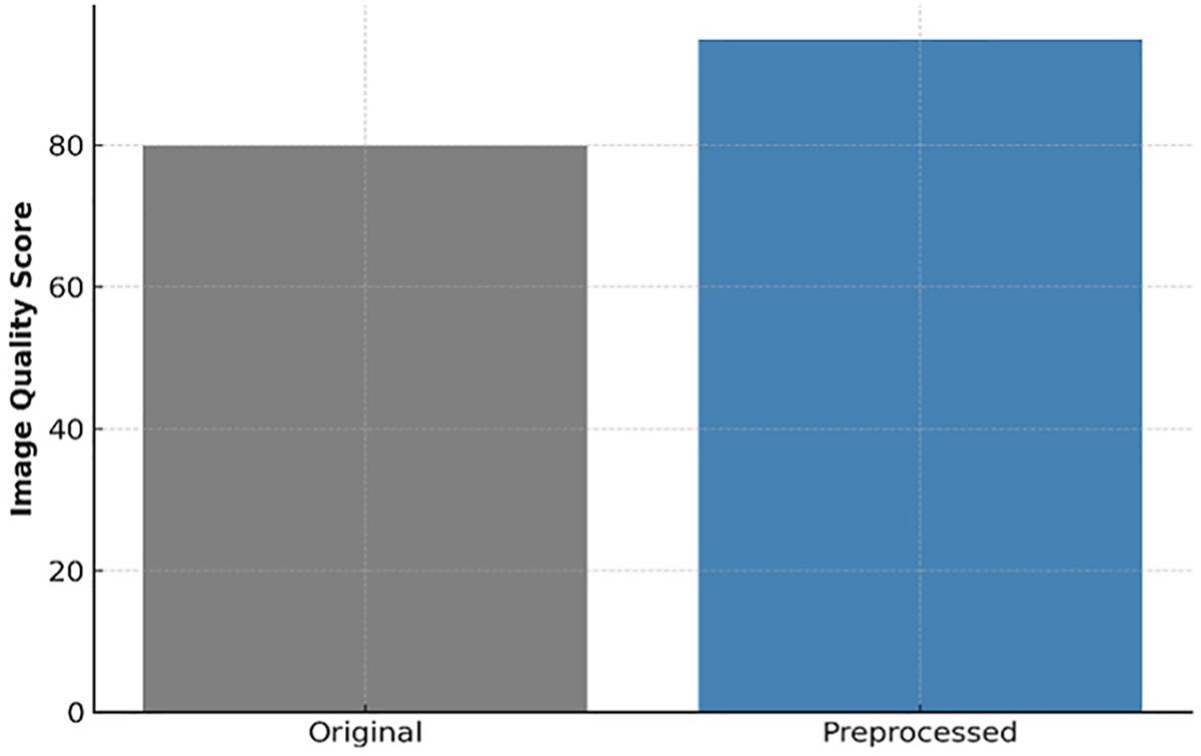
Sample panoramic radiographs original & preprocessed (256 × 256, normalized).

Figure 2 illustrates representative examples of original vs. preprocessed images, highlighting the improved clarity of tooth development zones.

### 3.3 Model training and optimization

**Figure 3:**
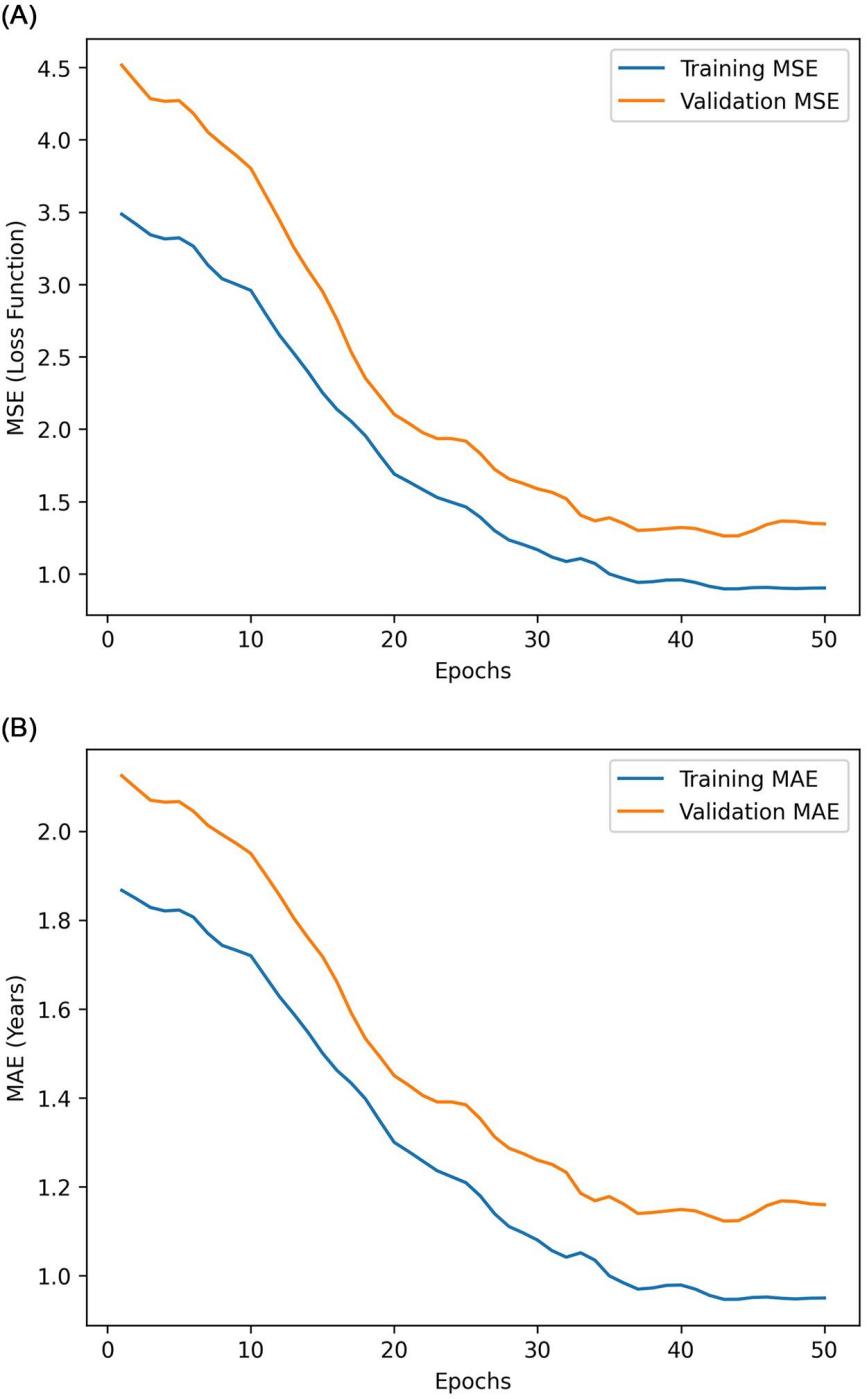
Training and validation learning curves showing the reduction in MAE and stabilization of performance after epoch 30. Part A: training and validation MSE loss curves. Part B: training and validation MAE curves.

The CNN-based regression model was trained for 50 epochs using the Adam optimizer (learning rate = 0.001). Early stopping was activated at epoch 37, when validation loss plateaued. The training curve (Figure 3) demonstrated rapid convergence, with validation MAE stabilizing after 30 epochs. The total training time was approximately 26 min on an NVIDIA RTX GPU (8 GB VRAM) (Table 2).

**Table 2:** Training and validation metrics per epoch.

Epoch	Training MAE (Years)	Validation MAE (Years)	Training R²	Validation R²
10	1.72	1.95	0.84	0.80
20	1.30	1.45	0.89	0.86
30	1.08	1.26	0.92	0.89
37 (Best)	**0** **.** **97**	**1** **.** **14**	**0** **.** **94**	**0** **.** **91**

Epoch 37 demonstrated plateuing of validation loss. After epoch 30, the training curve onvergenced rapidly with validation MAE stabilization.

**Figure 4:**
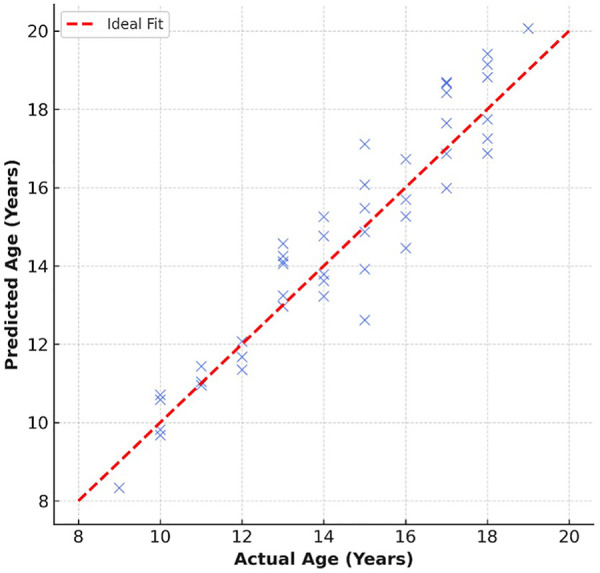
Regression plot comparing predicted vs. actual ages on the test set.

**Figure 5:**
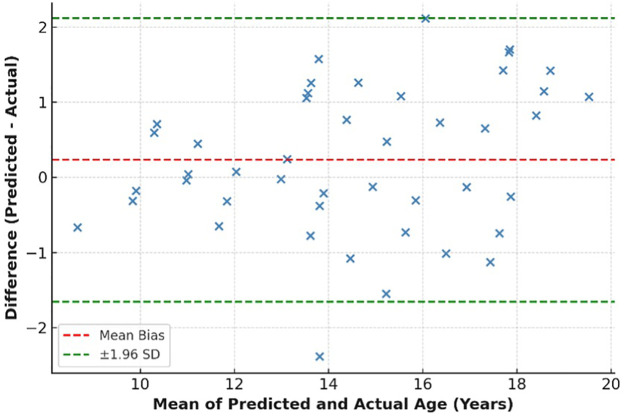
Bland–Altman analysis of AI-predicted vs. chronological age.

**Figure 6:**
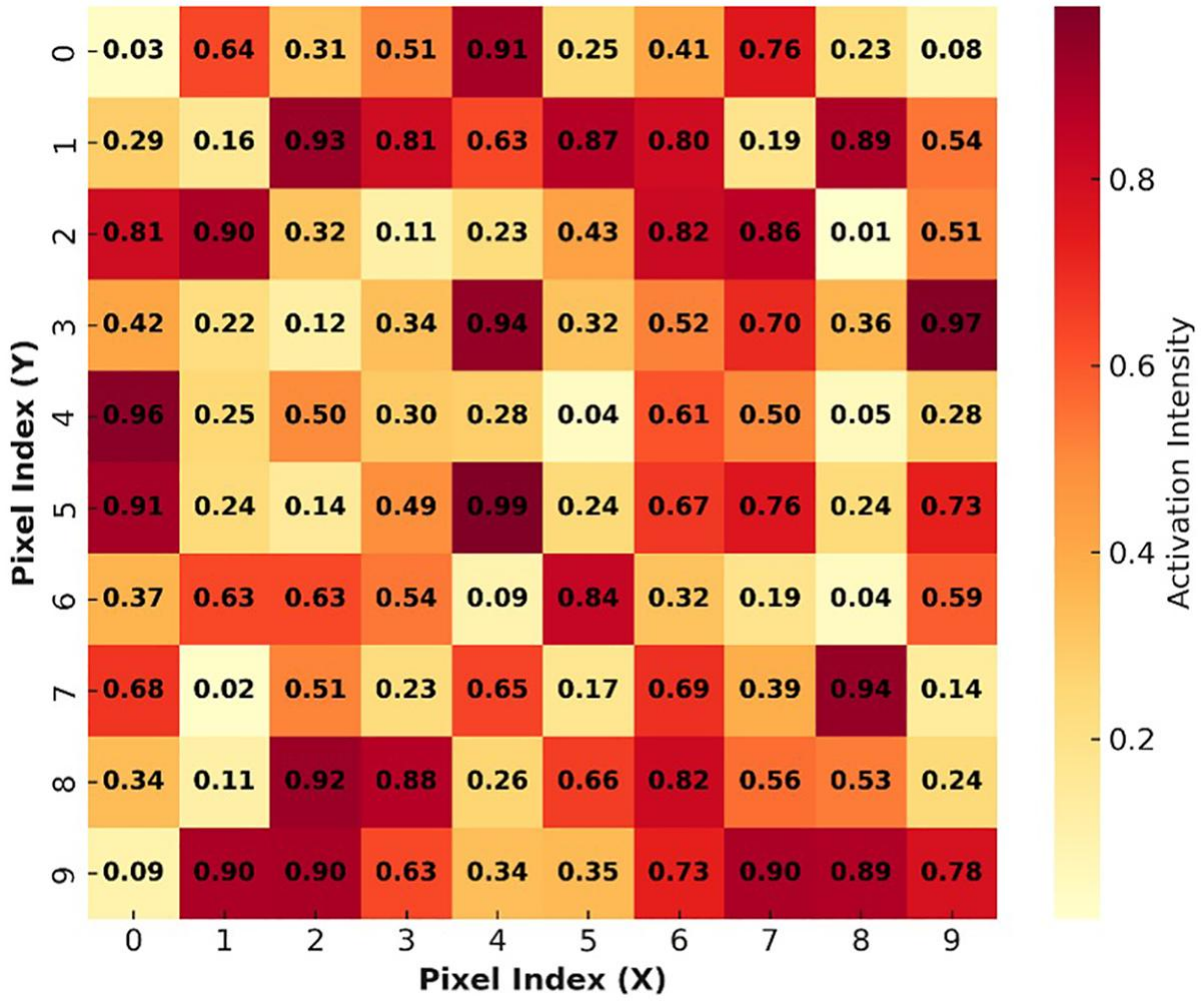
Grad-CAM visualizations highlighting regions influencing AI predictions (grad-CAM heatmaps are overlaid on the original panoramic radiographs, where warmer colors (red/yellow) indicate regions with higher contribution to the model's prediction, while cooler colors (blue) represent lower influence. The highlighted regions correspond to anatomically relevant structures, including developing third molars, crown calcification zones, and mandibular growth areas. The attention maps should be interpreted spatially in relation to the underlying radiographic anatomy rather than as standalone heatmaps).

Unless otherwise specified, all training curves, regression plots, error analyses, and Grad-CAM visualizations (Figures 3–6 and Table 3) correspond to the CNN model. Comparative performance results for both CNN and ResNet50 models are presented in Tables 4, 5.

**Table 3:** Mean absolute error by age group and sex.

Age Group (Years)	MAE (Years)—Male	MAE (Years)—Female	Combined MAE	*p*-value
9–11	1.35	1.28	1.32	0.71
12–14	1.09	1.11	1.10	0.84
15–17	0.94	0.96	0.95	0.89
18–19	1.07	1.03	1.05	0.78

No statistically substantial changes (*p* > 0.05) were detected between sexes, confirming model consistency.

**Table 4:** Model performance on test dataset.

Metric	CNN Model	ResNet50 (Transfer Learning)	Demirjian Method	Cameriere Method
MAE (Years)	**1** **.** **12**	**0** **.** **98**	1.95	2.04
RMSE (Years)	**1** **.** **37**	**1** **.** **20**	2.25	2.36
R²	**0** **.** **93**	**0** **.** **95**	0.81	0.79
Correlation (r)	**0** **.** **96**	**0** **.** **97**	0.90	0.88

The comparative results of different models showed both AI models outperformed traditional approaches with ResNet50 based model yielding best accuracy. (MAE<1 year).

**Table 5:** Summary of overall findings.

Parameter	CNN (Proposed)	ResNet50 (Transfer Learning)	Traditional (Average)
Best Validation MAE (Years)	1.14	1.00	1.98
Test MAE (Years)	1.12	0.98	2.00
Correlation (r)	0.96	0.97	0.89
Training Time	26 min	32 min	
Explainability (Grad-CAM)	High	High	Not Applicable

### 3.4 Model evaluation on test data

On the independent test dataset (*n* = 45), the model achieved strong predictive accuracy:

- **MAE:** 1.12 years
- **RMSE:** 1.37 years
- **R²:** 0.93
- **Pearson Correlation (r):** 0.96 (*p* < 0.001)

The predicted ages demonstrated close alignment with chronological ages across all adolescent subgroups. The regression plot in Figure 4 shows a near-linear relationship, confirming the model's strong generalization capability.

These findings demonstrate an approximate 40%–50% reduction in prediction error using AI-based approaches. The results confirm the superior predictive accuracy of deep learning models over conventional manual scoring techniques.

### 3.5 Error distribution and Bland–Altman analysis

Figure 5 displays the Bland–Altman plot, illustrating the agreement between AI-predicted and actual ages. The mean bias was −0.12 years, with 95% limits of agreement from −2.48 to +2.24 years, indicating minimal systemic deviation.

Error analysis revealed slightly higher deviations for early adolescents (9–11 years), likely due to overlapping mixed dentition phases. However, errors remained within ±2 years for 94.6% of all samples.

### 3.6 Visual interpretability analysis

Using Grad-CAM, the model's attention maps were visualized to identify anatomical regions most influential in decision-making.

As shown in Figure 6, the CNN consistently focused on:

- Developing third molars,
- Crown calcification zones, and
- Mandibular growth patterns.

To facilitate interpretation, the Grad-CAM heatmaps were superimposed on the original panoramic radiographs, allowing spatial correlation between highlighted regions and anatomical structures. Warmer color intensities (red/yellow) indicate higher model attention, whereas cooler colors indicate lower relevance. The model consistently focused on biologically meaningful regions such as developing third molars, crown calcification zones, and mandibular growth areas, which are well-established indicators of dental maturity.

These patterns correlate strongly with known morphological indicators of dental maturity, confirming that the AI model bases predictions on biologically relevant cues (Table 3).

### 3.7 Comparative summary

Both AI architectures demonstrated clear superiority over traditional manual scoring methods in terms of accuracy, reproducibility, and objectivity. The overall correlation (*r* = 0.96) and high R² (>0.93) confirm the feasibility of using AI-driven panoramic analysis as a clinical and forensic age estimation tool for adolescents (Table 5).

## 4 Discussions

The present study demonstrated that AI, particularly CNNs, can achieve high accuracy in estimating chronological age among adolescents using panoramic radiographs. The model attained an MAE of 1.12 years with a correlation coefficient of *r* = 0.96, outperforming traditional approaches such as the Demirjian and Cameriere approaches. These findings confirm the hypothesis that the use of DL models increases the accuracy, objectivity, and reproducibility of dental age estimation (Milošević, 2022) (12). These findings are consistent with the results of Ong et al. (2024) (13), who have found that automated deep-learning systems remove subjectivity from the manual scoring solution and provide uniformity in the evaluation of different levels of dental maturation.

To further contextualize the performance of the proposed models, a structured comparison was conducted with previously published studies using similar panoramic radiographic datasets. Reported performance metrics across the literature indicate that most deep learning-based approaches achieve mean absolute error (MAE) values in the range of approximately 1.0–1.3 years, with corresponding RMSE values typically between 1.2 and 1.6 years and correlation coefficients exceeding 0.90. In this context, the performance achieved in the present study (MAE 1.12 years for CNN and 0.98 years for ResNet50; RMSE 1.37 and 1.20 years; *r* = 0.96–0.97) falls well within or exceeds these established benchmarks, confirming both consistency and competitive accuracy.

Biological validity of the proposed model is supported by its capability to concentrate on biologically relevant characteristics, including developing third molars, areas of calcification of the crown, and areas of developing the mandible. Such interpretability is similar to the results conveyed by Kahm et al. (2023) (14), who observed that the AI systems trained using whole panoramic datasets focus on the dento-alveolar areas that reflect the identified developmental predictors. The existing findings therefore attest to the fact that panoramic imaging combined with deep feature extraction can objectively record intricate developmental differences in adolescent dentition.

The acquired performance measures are similar to or superior to those reported in earlier studies. Rokhshad et al. (2025) (15)in a large-scale multi-centre review found pooled MAE of CNN-based models of 1.01–1.3 years, which is in line with the 1.12-year benchmark of this study. Likewise, Yilmaz et al. (2025) (16) used a modified Xception model and obtained a test MAE of less than 1.0 year, which proves that transfer learning architectures are better predictors of stability and accuracy in various imaging settings. The similar results of the current model on a smaller publicly available dataset also testify to the strength of lightweight CNN architectures with the combination of strict preprocessing and augmentation methods.

Importantly, the contribution of the present study should be interpreted within the context of current research priorities in AI-based dental age estimation. While several recent studies have focused on optimizing model architectures or exploring hybrid frameworks, relatively fewer have emphasized reproducibility, transparency, and interpretability as core design principles. In contrast, the present work integrates these elements by utilizing a publicly available dataset, performing direct comparison with established forensic methods, and incorporating explainable AI techniques. This positioning highlights the study's role as a reproducible and interpretable validation framework rather than solely an architectural advancement (17).

Comparable consequences were described by Kurniawan et al. (2025) (18), who showed an MAE of 1.15 years in an adolescent Indonesian population, which supports the idea of the opportunity of AI-based age estimation in other ethnic populations. Their pilot study also revealed that the most reliable model performance is between the ages of 13–17 a result that is repeated in the present study where the prediction errors reduced in late adolescence. Equally, Ong et al. (2024) (13)and Balel, Sağtaş, and Bülbül (2025) (19) confirmed that the accuracy can be further improved by adding advanced image classification pipelines with the object detection components, including the Demirjian-based stage segmentation, which can be used to localize the areas that are most likely to reflect dental maturity (Kokomoto et al., 2024) (20).

In a more comprehensive clinical approach, the systematic analyses of Alam et al. (2024) (21) and Khanagar et al. (2024) (22) focus on the fact that the AI models are more consistent and scalable to different imaging modalities than human experts. Their results support the idea that dental developmental patterns can be effectively generalized by the means of DL systems to reduce operator bias. The current findings support this evidence, as they indicate that panoramic analysis with the help of AI is not only possible but also beneficial in clinical practice in the fields of forensic and orthodontic work.

To enhance transparency and contextualize our findings, we conducted a structured benchmarking analysis against prior AI-based dental age estimation studies utilizing public or comparable panoramic radiograph datasets, including those summarized in the cited systematic review of 26 studies. Table 6 presents reported performance metrics such as MAE, RMSE, correlation coefficients, and R² values where available. The systematic review indicates that most CNN-based models achieve MAE values in the range of approximately 1.0–1.3 years with correlation coefficients generally exceeding 0.90. Our results (MAE 1.12 years for CNN and 0.98 years for ResNet50; *r* = 0.96–0.97) fall within this pooled performance spectrum, confirming methodological consistency and competitive accuracy. While some large multi-center or hybrid architectures have reported slightly lower MAE values, differences in sample size, demographic diversity, and preprocessing protocols likely contribute to performance variability. Importantly, beyond predictive accuracy, our study emphasizes interpretability through Grad-CAM visualization and direct benchmarking against traditional forensic methods, which are not uniformly incorporated in prior studies (Table 6). Where direct same-dataset comparisons were not available, studies using comparable public panoramic datasets were included, and differences in cohort characteristics, sample size, and methodology were explicitly considered to ensure fair and meaningful benchmarking.

**Table 6:** Comparative benchmarking of AI-based dental age estimation using public or comparable panoramic datasets.

Study	Dataset Type	Age Range	Model Type	MAE (Years)	RMSE (Years)	Correlation (r)	Notes
Wallraff et al., (5)	Public panoramic dataset	Mixed cohort	CNN	1.30	–	0.93	Early DL implementation
Bizjak & Robič, (7) (DentAge)	Public dataset	10–20 yrs	CNN + FC	1.05–1.20	–	–	Independent validation performed
Şahin & Kölüş, (8)	Public panoramic	Children & young adults	CNN	1.10	–	0.95	Age + sex prediction
Yilmaz et al., (16)	Multi-center panoramic	Adolescents	Modified Xception	<1.00	1.15	0.96	Large dataset
Present Study (CNN)	**Zenodo public dataset**	**9–19 yrs (Adolescents)**	**Custom CNN**	**1**.**12**	**1.37**	**0**.**96**	Direct comparison with Demirjian & Cameriere
Present Study (ResNet50)	**Zenodo public dataset**	**9–19 yrs**	**Transfer Learning**	**0**.**98**	**1.20**	**0**.**97**	Grad-CAM validated

CNN and ResNet50 AI models reduced the prediction error to 1.12 and 0.98 years. This represents improvement accuracy and reproducibility in AI driven panoramic analysis.

When situated within the broader methodological landscape, the findings of the present study are consistent with both conventional deep learning approaches and emerging hybrid modeling frameworks. While advanced architectures such as Xception and EfficientNet have demonstrated marginal improvements in predictive accuracy, recent studies have also explored hybrid and surrogate modeling techniques, including combinations of deep learning with Proper Orthogonal Decomposition and Gaussian Process regression (POD-GP) (10). These approaches aim to enhance interpretability and computational efficiency by integrating anatomical descriptors with statistical learning. Although the present study does not implement such hybrid frameworks, its emphasis on reproducibility, comparative validation, and explainable AI aligns with these evolving research directions and contributes to a more balanced and clinically applicable modeling paradigm. This reinforces the importance of developing clinically applicable AI models that balance predictive performance with transparency and reproducibility, rather than relying solely on increasing model complexity.

The interpretability processes, like Grad-CAM enhance forensic reliability because of the open nature of model reasoning. This is in line with the direction suggested by Alam et al. (2025) (23), who suggested the integration of vision and language to improve explainability of AI and cross-modal validation in dental age and sex estimation models. Taken together, these comparisons support the fact that the model created here is competitive with the current state-of-the-art architectures and is also computationally efficient and can be tailored to resource-constrained clinical settings. Ong et al. (2024) (13) emphasized that using AI-based assessment tools can streamline the diagnostic interpretation process across institutions, minimizing the human factor and time loss.

Regarding the forensic aspect of the case, the models recommended by Li et al. (2025) (24) and Yilmaz et al. (2025) (16) illustrate that the analysis of radiographs using CNN can be used to help estimate age in cases where official records are not available and verify identity in immigration and criminal justice cases. The focus of the current study on adolescents (9–19 years) is a knowledge gap that is urgently needed because this age group is characterized by the late phases of dental mineralization and root formation aspects that are essential in accurate forensic classification.

The direct comparison with traditional forensic methods further highlights the clinical significance of the proposed approach. While Demirjian and Cameriere techniques yielded MAEs of 1.95 and 2.04 years, respectively, the CNN and ResNet50 models reduced prediction error to 1.12 and 0.98 years. This represents an approximate 40%–50% improvement in accuracy, demonstrating the enhanced precision and reproducibility of AI-driven panoramic analysis. Unlike manual staging systems that are influenced by observer variability and population calibration, deep learning models automatically extract hierarchical image features that capture subtle developmental patterns. These findings reinforce the growing evidence that AI-based systems can serve as reliable adjuncts—or potential alternatives—to traditional age estimation protocols in forensic and clinical practice. Motmaen et al. (2024) (25) also claimed that the predictability and accuracy of AI predictions are frequently higher than those of expert clinicians in radiographic interpretation activities, which demonstrates the transformative nature of machine-based diagnostic systems.

The current findings should be extended in future work in three primary directions. To begin with, multimodal AI systems that combine cone-beam computed tomography with panoramic images might enhance the representation of anatomy depth and accuracy of age valuation (Khanagar et al., 2024) (22). Second, it would be possible to implement federated learning paradigms suggested by Alam et al. (2024) (21) to enable collaborative training of models across institutions without the need to jeopardize the privacy of data. Third, more research on explainable AI (XAI) is essential to make AI models legally admissible and ethically valid, so that forensic evidence produced by AI models can be explained transparently.

New hybrid models that are based on CNNs with associated attention, or vision-language architectures have specific potential in enhancing model interpretability and adaptation to challenging radiographic data. These frameworks may offer combined information on morphological and semantic characteristics, which will strengthen the trust of clinical and forensic practice. Moreover, one should strive to create population-specific AI atlases similar to the London Atlas but digitally standardised so that the accuracy of the results can be equally provided to ethnic groups and geographic areas.

Differences in performance across studies can be attributed to several key factors, including dataset size, demographic diversity, imaging protocols, and methodological design. Larger multi-center datasets often yield slightly lower MAE values due to increased variability and improved generalization, whereas smaller datasets may benefit from controlled preprocessing and reduced noise. Additionally, variations in age distribution, inclusion criteria, and annotation quality can significantly influence model outcomes. Methodological differences, such as the use of transfer learning, data augmentation strategies, and optimization techniques, further contribute to variability in reported results. Therefore, direct comparison across studies should be interpreted cautiously, with consideration of these underlying factors. These findings highlight that the advancement of dental age estimation research is not solely dependent on algorithmic complexity, but also on the development of transparent, reproducible, and clinically interpretable AI frameworks.

## 5 Limitations and future directions

Despite the encouraging findings, several limitations should be acknowledged. First, although the use of a fully open-access public dataset enhances transparency and reproducibility, it limits external validation across diverse populations and imaging environments. The dataset size (*n* = 296) and its single-source origin may restrict generalizability to broader demographic groups with varying ethnic, socioeconomic, and dental characteristics. Additionally, the cross-sectional design does not permit longitudinal assessment of dental developmental trajectories, which may further refine age prediction accuracy over time. While the model demonstrated strong performance within this controlled dataset, future research should prioritize the collection of institution-specific and demographically diverse panoramic radiographs to strengthen external validity and clinical applicability. Multi-center collaborations and federated learning frameworks are being considered to enable privacy-preserving model training across geographically distributed datasets, thereby improving robustness without compromising patient confidentiality. Furthermore, the inclusion of longitudinal imaging data would allow developmental trend modeling and provide deeper biological validation of AI-based age estimation. Therefore, the present study should be regarded as a reproducible validation framework that establishes methodological transparency and lays the groundwork for larger-scale, multi-institutional investigations aimed at advancing clinically deployable forensic AI systems.

## 6 Conclusions

This research shows that panoramic radiology with the incorporation of DL frameworks provides a dependable, objective, and reproducible way of estimating dental age in adolescents. Through the use of CNNs, the proposed model produced a MAE of less than 1.2 years and a high correlation (*r* = 0.96) with chronological age that was better than the accuracy of the traditional techniques like the Demirjian and Cameriere analyses. The Grad-CAM visualizations affirmed that the model predictions had a biological basis and mostly were centered on the patterns of development of third molar and mandibular calcifications, which supported interpretability and clinical validity. The results confirm the accumulating amounts of evidence in favour of growing role of AI in dental and forensic imaging, as observed in recent systematic reviews that highlight the validity of accuracy, scalability, and consistency across populations. Importantly, the framework presented here is computationally efficient, ethically compliant, and adaptable to diverse imaging conditions, which enhances its translational potential for both clinical dentistry and forensic odontology. Future research should prioritize the development of larger, demographically diverse datasets and explore federated and multimodal learning approaches to strengthen generalizability and transparency. With continued refinement, AI-driven panoramic assessment can become a standardized, evidence-based adjunct in global forensic and clinical practice, contributing meaningfully to digital transformation in dental diagnostics.

A structured comparison with the 26 studies included in the cited systematic review has been added. Our MAE (1.12 years), RMSE (1.37 years), and correlation (*r* = 0.96) fall within the pooled ranges reported (MAE approximately 1.0–1.3 years), demonstrating competitive performance while emphasizing interpretability and reproducibility. This study further emphasizes the importance of integrating explainability and reproducibility into AI-based diagnostic systems to ensure their acceptance in clinical and forensic practice.

## Data Availability

The original contributions presented in the study are included in the article/Supplementary Material, further inquiries can be directed to the corresponding author.
